# Mirikizumab impact on disease clearance in patients with moderately to severely active ulcerative colitis: analysis of a pre-specified LUCENT trial endpoint

**DOI:** 10.1093/ecco-jcc/jjaf124

**Published:** 2025-07-13

**Authors:** Jean-Frederic Colombel, Jianmin Wu, Taku Kobayashi, Baojin Zhu, Jerome Paulissen, Agni Dhanabal, Vipul Jairath, Corey Siegel, Britta Siegmund, Isabel Redondo, Richard Moses, Fernando Magro

**Affiliations:** Henry D. Janowitz Division of Gastroenterology, Department of Medicine, Icahn School of Medicine at Mount Sinai, New York, NY, United States; Eli Lilly and Company, Indianapolis, IN, United States; Center for Advanced IBD Research and Treatment, Kitasato University Kitasato Institute Hospital, Tokyo, Japan; Eli Lilly and Company, Indianapolis, IN, United States; Syneos Health, Morrisville, NC, United States; Eli Lilly and Company, Indianapolis, IN, United States; Department of Medicine, Division of Gastroenterology, Western University, London, Ontario, Canada; Inflammatory Bowel Disease Center, Dartmouth-Hitchcock Medical Center, Lebanon, NH, United States; Department of Gastroenterology, Infectious Diseases and Rheumatology, Charité – Universitätsmedizin Berlin, corporate member of Freie Universität Berlin, Humboldt-Universität zu Berlin, Medizinische Klinik für Gastroenterologie, Infektiologie und Rheumatologie, Berlin, Germany; Medical Affairs, Eli Lilly and Company, Lisbon, Portugal; Eli Lilly and Company, Indianapolis, IN, United States; Center for Health Technology and Services Research (CINTESIS@RISE), Faculty of Medicine, University of Porto, Porto, Portugal; Faculty of Medicine, University of Porto and ULS São João, Porto, Portugal

**Keywords:** disease clearance, mirikizumab, ulcerative colitis

## Abstract

**Background and aims:**

Concurrent achievement of symptomatic, endoscopic, and histologic remission, known as disease clearance, has been proposed as a treatment target in ulcerative colitis. Mirikizumab, an anti-interleukin-23p19 antibody, has demonstrated long-term efficacy and safety, as reported in the LUCENT Phase 3 trials (NCT03518086, NCT03524092, and NCT03519945). The current analysis evaluates the impact of mirikizumab on disease clearance and its association with other clinical and patient-reported outcomes (PROs).

**Methods:**

LUCENT methods have previously been reported. The proportion of patients achieving disease clearance was determined through week (W)104. Association analyses were assessed between disease clearance and PROs (IBDQ, SF-36, WPAI: UC, PGRS, and PGRC), and early disease clearance and subsequent clinical outcomes (clinical, corticosteroid-free, endoscopic, histological, histologic-endoscopic, bowel urgency, stool frequency, and rectal bleeding).

**Results:**

The proportions of patients achieving disease clearance with mirikizumab at W12, W52, and W104 were 16.0%, 36.4%, and 51.3%, respectively. Mirikizumab-treated patients with disease clearance showed greater PRO improvements through W52 than those without. Early clearance at W12 was associated with significantly better clinical outcomes at later time points, except for bowel urgency remission at W52. This trend was repeated for clinical outcomes at W104 in patients who achieved disease clearance at W52.

**Conclusions:**

Mirikizumab consistently demonstrated disease clearance across induction, maintenance, and long-term studies. The attainment of disease clearance was associated with greater improvement in PROs, and early achievement of disease clearance was associated with better long-term outcomes, including clinical remission, corticosteroid-free remission, endoscopic and histological outcomes, reduced stool frequency, and rectal bleeding.

## 1. Introduction

In the search for optimal therapeutic strategies for ulcerative colitis (UC), disease clearance (DC)—the concurrent achievement of symptomatic, endoscopic, and histologic remission—has been proposed as an aspirational target, a shift from traditional treatment goals.^1–3^ In the past, the primary focus of UC management has been symptom relief, sometimes neglecting gut healing and complete remission. This limitation has hindered long-term disease control and outcomes.

The specific benefits of achieving DC in the treatment of UC could include improved disease course, better long-term outcomes, enhanced quality of life (QoL), and predictive value regarding the efficacy of treatment(s).^1^ Patients with DC have a lower risk of disease recurrence, hospitalization, and surgery, leading to better overall control of UC and improved QoL.^4^ Additionally, histologic remission, a component of DC, is predictive of sustained clinical remission and lower relapse rates.^5^ Thus, achieving DC may improve the management and prognosis of UC. Additionally, data from an Australian multicenter retrospective study indicated that 61% of patients achieved clinical remission, of whom 57% also showed endoscopic remission. In total, 35% of these patients showed combined clinical and endoscopic remission, and 16% had combined clinical, endoscopic, and histologic remission.^6^ Together these results demonstrate that DC in UC is an achievable goal.

The introduction of mirikizumab, a novel anti-interleukin-23p19 monoclonal antibody, has marked an advancement in the treatment landscape of UC. Empirical evidence from the LUCENT Phase 3 trials underscores the robust efficacy of mirikizumab, as evidenced by its impact on individual and composite clinical endpoints.^7–9^ However, traditional endpoints may not fully encapsulate the comprehensive benefits of achieving DC.

This analysis examines the effects of mirikizumab on achieving DC across 2 years within the LUCENT trials. It also investigates the association between DC and a range of clinical and patient-reported outcomes (PROs), offering a more detailed understanding of the drug’s impact on DC and its associated effect on other clinical outcomes.

## 2. Methods

### 2.1. LUCENT-1, -2, and -3 study overviews

The LUCENT-1, LUCENT-2, and LUCENT-3 study designs have been reported previously.^7^^,^^9^ These studies comprised 2 sequential, parallel-group, double-blind, randomized, placebo (PBO)-controlled trials, followed by an open-label long-term extension in adult patients with moderately to severely active UC who had an inadequate response or loss of response to, or were unable to, tolerate at least 1 of the following: corticosteroids, 6-mercaptopurine, azathioprine, biologic therapy (tumor necrosis factor inhibitors or vedolizumab), or tofacitinib. LUCENT-1 was a 12-week induction study, followed by the 40-week LUCENT-2 randomized withdrawal maintenance study in patients who responded to mirikizumab induction therapy in LUCENT-1. LUCENT-3 included patients from the phase 3 maintenance study LUCENT-2 who completed the Week (W)52 (W40 LUCENT-2) visit on blinded subcutaneous therapy and, per investigator opinion, would benefit from continuing treatment with mirikizumab in LUCENT-3 (Figure S1).

The studies were conducted in compliance with the consensus ethics principles derived from international ethics guidelines, including the Declaration of Helsinki, the International Council for Harmonisation, and applicable laws and regulations. All patients provided written informed consent before participating in the study. An independent data monitoring committee monitored LUCENT-1, LUCENT-2, and LUCENT-3. The trials were registered at Clinical Trials.gov: NCT03518086, NCT03524092, and NCT03519945, respectively.

### 2.2. Study assessments

The primary and major secondary endpoints for LUCENT-1 and LUCENT-2 have been previously reported.^7^^,^  ^10^ The baseline data for the current analyses were the induction baseline data from LUCENT-1. The endpoint of DC and related analyses were pre-specified in the LUCENT trial Statistical Analysis Plan. The definition of DC and other endpoints associated with the current analyses can be found in Table 1. It is important to note that the Patient Global Rating of Change (PGRC) ranges from 1 to 7, where 1 equals “very much better” (symptoms) and 7 equals “very much worse”; thus, a lower PGRC reflects symptom improvement.^11^ Patient Global Rating of Severity (PGRS) assesses disease symptom severity of patients over the past 24 hours using a 6-point scale where 1 equals “none” (symptoms) to 6 equals “very severe symptoms”. DC achievement was further evaluated based on the concurrent achievement of symptomatic remission, histologic-endoscopic mucosal remission (HEMR), and fecal calprotectin (fCal) <250 μg/g (alternative DC [aDC]).

**Table 1.** Endpoints associated with the current analyses.

Disease clearance	
** Disease clearance (DC)**	Disease clearance defined as symptomatic remission and HEMR.
** Alternative DC**	Disease clearance defined as symptomatic remission, HEMR, and fecal calprotectin <250 μg/g.
**Clinical endpoints**	
** Clinical remission**	Modified Mayo score (MMS) Stool Frequency subscore (SF) = 0 or SF = 1 with a ≥ 1-point decrease in MMS SF from baseline; Rectal bleeding (RB) = 0; and MMS endoscopic subscore (ES) = 0 or ES = 1 (excluding friability).
** Symptomatic remission**	SF = 0 or SF = 1 with a ≥ 1-point decrease in MMS SF from baseline; RB = 0.
** Endoscopic remission**	ES = 0 or 1 (excluding friability).
** Histologic remission**	Geboes scoring of ≤2B.0 (Geboes subscores of 0 for grades 2 b [lamina propria neutrophils], 3 [neutrophils in epithelium], 4 [crypt destruction], 5 [erosion or ulceration]).
** Corticosteroid-free remission**	Week (W)52 (LUCENT-2): W52 clinical remission, W40 symptomatic remission, and no corticosteroid (CS) use ≥12 weeks before W52. W104 (LUCENT-3): W104 clinical remission and no CS use ≥12 weeks before W104.
** Histologic-endoscopic mucosal remission (HEMR)**	Geboes scoring of ≤2B.0 (Geboes subscores of 0 for grades 2 b [lamina propria neutrophils], 3 [neutrophils in epithelium], 4 [crypt destruction], 5 [erosion or ulceration]) and ES = 0 or ES = 1 (excluding friability).
** Bowel urgency remission**	Urgency numeric rating scale (NRS) score = 0 or 1.
**Stool frequency remission**	MMS SF = 0 with a decrease of ≥1-point from induction baseline.
** Rectal bleeding remission**	MMS RB = 0.
**Patient-reported outcomes**	
** Fatigue numeric rating scale (NRS)**	11-point NRS scale (0 = no fatigue to 10 = fatigue as bad as you can imagine; change from baseline).^11^
** Inflammatory Bowel Disease Questionnaire remission (IBDQ)**	7-point Likert scale (7 = not a problem at all to 1 = a very severe problem, total score ≥170 indicates disease remission).^24^
** 36-Item Short Form Survey (SF-36)**	Scores of 0–100 (higher scores indicate better health or function). Comprises physical component summary and mental component summary components (change from baseline).^12^
** Work Productivity and Activity Impairment Questionnaire: Ulcerative Colitis (WPAI: UC)**	WPAI: UC scores (absenteeism, presenteeism, work productivity loss in employed patients, and activity impairment in all patients), ranging from 0 to 100 (higher scores indicate greater limitation).^13^
** Patient’s Global Rating of Severity (PGRS)**	1-item patient-rated questionnaire designed to assess patients’ rating of their disease symptom severity over the past 24 hours using a 6-point scale (1 = none to 6 = very severe).
** Patient’s Global Rating of Change (PGRC)**	Patient-rated instrument designed to assess patients’ rating of change in their symptom(s) using a 7-point Likert scale (1 = very much better to 7 = very much worse).

### 2.3. Statistical analyses

The assessment of DC among patients was conducted at W12, W52, and W104. The modified intent-to-treat population, including all patients who received any study treatment during this study and excluding patients impacted by an electronic clinical outcome assessment transcription error in Poland (*n *= 112) and Turkey (*n *= 6) (see D’Haens et al., 2023 Supplementary Appendix for details^7^), regardless of whether the patient received the correct treatment or did not otherwise follow the protocol, was used to perform efficacy analyses at the indicated time points. Subgroup analyses were conducted based on the following subgroups: patients with no prior biologic or tofacitinib treatment exposure (bio-naïve) and patients with prior biologic or tofacitinib treatment failure (bio-failed). Prior biologic or tofacitinib treatment failure was defined as discontinuation of prior treatment due to loss of response, inadequate response, or intolerance to the therapy.

In the modified intent-to-treat population, treatment comparisons for binary outcomes involved a Cochran–Mantel–Haenszel (CMH) test adjusted for randomization factors (prior biologic or tofacitinib failure [yes/no], baseline corticosteroid use [yes/no], baseline disease activity assessed by the modified Mayo score [MMS, 4–6 or 7–9], and region [North America/Europe/Other]) for LUCENT-1. For LUCENT-2, the adjustment included all the above factors except the modified Mayo score, and additionally included clinical remission status at LUCENT-1 W12 (yes/no).

The associations between PROs and DC were evaluated for patients treated with mirikizumab at W12 and W52, respectively. A CMH test was also used to assess associations between DC and binary outcomes, while analysis of covariance with modified baseline observation carried forward was used to assess the association of DC with continuous outcomes. The associations between DC at W12 and baseline factors, and between DC at W52 and baseline factors, were evaluated using univariable and multivariable logistic regression analyses. Variables with *P* <.05 in the univariable models were included in the multivariable analyses. Moreover, the association between early achievement of DC and subsequent long-term clinical outcomes was evaluated in mirikizumab-treated patients. Specifically, DC at W12 was assessed for its relationship with clinical outcomes at W52, and DC at W52 with outcomes at W104. Pearson’s chi-square test was used to evaluate these associations, with odds ratios and 95% confidence intervals provided to quantify the strength of the relationships.

Nonresponder imputation (NRI) was used for all binary outcome analyses, including DC (yes/no). Observed Case (OC) analysis was also performed in some supplemental results at W104 to assess the proportion of mirikizumab-treated patients achieving DC among W52 clinical responders. The methods have been described previously in OC analyses, patients with missing data were excluded, and no imputation was applied.

## 3. Results

### 3.1. Achievement of DC during induction, maintenance, and long-term treatment

**Figure 1.**
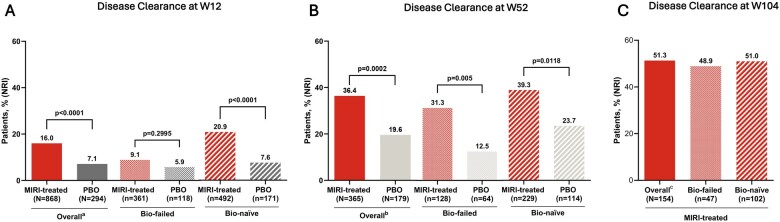
(A) The proportion of patients achieving DC at W12 in LUCENT-1. (B) The proportion of patients achieving DC at W40 in LUCENT-2 (52-week treatment in total). (C) Proportion of patients achieving DC at W104 among patients who achieved clinical remission at W52 and subsequently entered the LUCENT-3. ^a^5 patients receiving PBO and 15 patients receiving MIRI were previously exposed to but had no biologic or JAK inhibitor failure and were excluded from the biologic subgroup analysis. ^b^1 patient receiving PBO and 8 patients receiving MIRI were previously exposed to but had no biologic or JAK inhibitor failure and were excluded from biologic subgroup analysis. ^c^5 patients were previously exposed to but had no biologic or JAK inhibitor failure and were excluded from the biologic subgroup analysis. DC = symptomatic remission + histologic-endoscopic mucosal remission. DC, disease clearance; JAK, Janus kinase; MIRI, mirikizumab; NRI, nonresponder imputation; PBO, placebo; W, Week.

In the modified intent-to-treat population, a significantly greater proportion of mirikizumab-treated patients achieved DC versus placebo at W12 (16.0% vs 7.1%; *P* <.0001; Figure 1A). At W52, 36.4% of patients treated with mirikizumab achieved DC versus 19.6% with placebo (*P* <.001). Overall, a higher proportion of patients treated with mirikizumab achieved DC compared to those receiving placebo, in both the bio-naïve and bio-failed subgroups at Weeks 12 and 52. Notably, in the bio-failed subgroup at W52, 31.3% of patients receiving mirikizumab achieved DC versus 12.5% in the placebo group. Similarly, in the bio-naïve subgroup, 39.3% of patients on mirikizumab achieved DC compared to 23.7% with placebo (Figure 1B). About 51.3% of patients on mirikizumab achieved DC at W104 among those who achieved clinical remission at W52, and the rates of patients achieving DC were similar to those of the overall population in both the bio-failed (48.9%) and bio-naïve (51.0%) subgroups (Figure 1C). Results were similar when assessing patients with a baseline modified Mayo Score (mMS) of 5–9 (excluding patients with baseline mMS of 4) per updated FDA guidelines (Figure S2).^12^ DC achievement in the long-term study was also explored in patients who achieved clinical response on mirikizumab (W52 clinical responders). About 40.6% of the W52 clinical responders achieved DC at W104 (Figure S3). Among patients who achieved DC at W12 with mirikizumab induction, completed LUCENT-2 on mirikizumab 200 mg Q4W subcutaneous and enrolled in LUCENT-3 (*N *= 69, NRI), 52.2% achieved clinical remission, 50.7% achieved CSF remission, 52.2% achieved HEMR, and 75.4% achieved IBDQ remission at W104.

When including assessment of fecal calprotectin in the composite DC endpoint, alternative DC (aDC), a higher proportion of mirikizumab-treated patients achieved aDC at W12 (13.1% vs 4.4%; *P* <.0001) and W52 (31.0% vs 16.8%; *P* = .0013; Figure S4). This effect was significant in the bio-naïve subgroup at both timepoints, and in the bio-failed subgroup at W52. In addition, a significantly higher proportion of patients in the bio-naïve subgroup on mirikizumab achieved aDC at both timepoints.

### 3.2. Association between DC achievement and PROs

**Figure 2.**
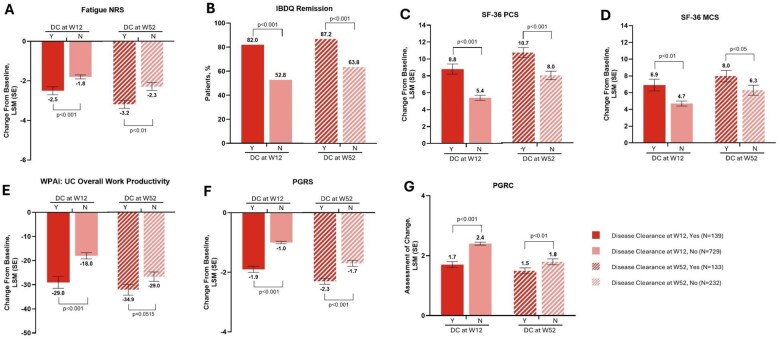
Association between achievement of DC and patient-reported outcomes in mirikizumab-treated patients; fatigue NRS (a), IBDQ remission (B), SF-36 PCS (C), SF-36 MCS (D), WPAI: UC overall work productivity (E), PGRS (F), and PGRC (G) at W12 and W52. DC, disease clearance; IBDQ, inflammatory bowel disease questionnaire; LSM, least-squares mean; MCS, mental component summary; N, no; NRS, numeric rating scale; PCS, physical component summary; PGRC, patient global rating of change; PGRS, patient global rating of severity; SF-36, 36-item short form health survey; UC, ulcerative colitis; WPAI, work productivity and activity impairment questionnaire.

The change in Fatigue numeric rating scale (NRS) score, Work Productivity and Activity Impairment Questionnaire: Ulcerative Colitis (WPAI: UC), Overall Work Productivity, Patient Global Rating of Severity (PGRS), and PGRC from baseline and achievement of PROs (Inflammatory Bowel Disease Questionnaire [IBDQ] Remission, 36-Item Short Form Survey [SF-36] physical component summary, and SF-36 mental component summary) at W12 and W52 were compared based on achievement of DC (yes vs no) with mirikizumab at W12 and W52, respectively (Figure 2). Patients who achieved DC at W12 had significantly higher rates of achievement of PROs and larger improvements from baseline of PROs versus patients who did not achieve DC (all *P* <.01). The trend continued among patients who achieved DC at W52 versus those who did not, but this trend was not seen for WPAI: UC Overall Work Productivity (*P* = .0515). While not statistically significant, there was a larger improvement in WPAI: UC Overall Work Productivity at W52 among those who achieved DC at W52 than among those who did not (change from baseline of −34.9 vs −29.0).

### 3.3. Association of early achievement of DC and subsequent achievement of long-term positive clinical outcomes

Achievement of DC at W12 was significantly associated with achievement of clinical remission, corticosteroid (CS)-free remission, endoscopic remission, HEMR, stool frequency (SF) remission, and rectal bleeding (RB) remission at W52 (*P* <.05; Table 2). Notably, achievement of DC at W12 was not associated with bowel urgency (BU) remission, IBDQ response, and IBDQ remission at W52. The trend continued where the achievement of DC at W52 was associated with the achievement of the same positive clinical outcomes, including CS-free remission, at W104 (*P* <.05; Table 3), except for SF and RB remission at W104 which were not significantly associated with DC at W52.

**Table 2.** Association of DC at week 12 and subsequent achievement of clinical outcomes in mirikizumab-treated patients at week 52.

Clinical outcome at Week 52	Achieved DC at Week 12 (*N *= 93)	Did not achieve DC at Week 12 (*N *= 272)	*P*-value	Odds Ratio (95% CI)
**Clinical remission**	59 (63.4)	123 (45.2)	<0.01	2.1 (1.3, 3.4)
**CS-free remission**	55 (59.1)	109 (40.1)	<0.01	2.2 (1.3, 3.5)
**Endoscopic remission**	64 (68.8)	150 (55.1)	<0.05	1.8 (1.1, 3.0)
**Histologic remission**	60 (64.5)	117 (43.0)	<0.001	2.4 (1.5 3.9)
**HEMR**	55 (59.1)	103 (37.9)	<0.001	2.4 (1.5, 3.8)
**BU remission**	44 (47.3)	114 (41.9)	0.365	1.2 (0.8, 2.0)
**SF remission**	79 (84.9)	195 (71.7)	<0.05	2.2 (1.2, 4.2)
**RB remission**	83 (89.2)	208 (76.5)	<0.01	2.6 (1.3, 5.2)
**IBDQ remission**	74 (79.6)	190 (69.9)	0.071	1.7 (1.0, 3.0)
**IBDQ response**	76 (81.7)	213 (78.3)	0.485	1.2 (0.7, 2.3)

Data are presented as *n* (%) unless otherwise stated. Mirikizumab induction responders treated with 200 mg subcutaneous mirikizumab in LUCENT-2 were included. *P*-values were derived from Pearson’s chi-square test.

Abbreviations: BU, bowel urgency; CS, corticosteroid; DC, disease clearance; HEMR, histologic-endoscopic mucosal remission; IBDQ, Inflammatory Bowel Disease Questionnaire; RB, rectal bleeding; SF, stool frequency.

**Table 3.** Association of DC at week 52 and subsequent achievement of clinical outcomes in mirikizumab-treated patients at week 104.

Clinical outcome at Week 104	Achieved DC at Week 52 (*N *= 111)	Did not achieve DC at Week 52 (*N *= 128)	*P*-value	Odds Ratio (95% CI)
**Clinical remission**	73 (65.8)	56 (43.8)	<0.001	2.5 (1.5, 4.2)
**CS-free remission**	71 (64.0)	55 (43.0)	<0.01	2.4 (1.4, 4.0)
**Endoscopic remission**	87 (78.4)	69 (53.9)	<0.001	3.1 (1.8, 5.5)
**Histologic remission**	71 (64.0)	53 (41.4)	<0.001	2.5 (1.5, 4.2)
**HEMR**	68 (61.3)	46 (35.9)	<0.001	2.8 (1.7, 4.8)
**BU remission**	58 (52.3)	62 (48.4)	0.557	1.2 (0.7, 1.9)
**SF remission**	82 (73.9)	87 (68.0)	0.318	1.3 (0.8, 2.3)
**RB remission**	85 (76.6)	88 (68.8)	0.178	1.5 (0.8, 2.6)
**IBDQ remission**	91 (82.0)	96 (75.0)	0.193	1.5 (0.8, 2.8)
**IBDQ response**	90 (81.1)	104 (81.3)	0.974	1.0 (0.5, 1.9)

Data are presented as *n* (%) unless otherwise stated. Mirikizumab W52 responders were included. *P*-values were derived from Pearson’s chi-square test.

Abbreviations: BU, bowel urgency; CS, corticosteroid; DC, disease clearance; HEMR, histologic-endoscopic mucosal remission; IBDQ, Inflammatory Bowel Disease Questionnaire; RB, rectal bleeding; SF, stool frequency.

### 3.4. Univariable and multivariable prognostic analyses of association between baseline demographics and characteristics, and DC

Baseline age, body mass index, disease duration, MMS, and prior UC therapies were similar between patients who achieved DC at W12 and those who did not (Table S1).

Associations between demographic and disease characteristics at induction baseline and DC at W12 were analyzed in patients randomized to mirikizumab 300 mg IV in LUCENT-1. Characteristics including left-sided colitis/proctitis, fCal ≤250 μg/g, C-reactive protein [CRP] ≤6 mg/L, baseline MMS, endoscopic subscore (ES) = 2, SF subscore <3, and no prior biologic or tofacitinib failure were significantly associated with DC at W12 in the univariable analysis (Table S2). In the multivariable model, CRP ≤6 mg/L, baseline MMS, ES = 2, SF subscore <3, and no prior biologic or tofacitinib failure were significantly positively associated with DC at W12 (Table S3). Univariable analysis showed that only baseline disease location was significantly associated with DC at W52 (*P* = .017). Since multivariable analysis selects significant variables from the univariable model and only 1 variable is significant for W52, no multivariable analyses for W52 were assessed (data not shown).

## 4. Discussion

This analysis demonstrates achievement of DC with mirikizumab treatment in the LUCENT trials based on stringent definitions of DC that include the concurrent achievement of symptomatic remission (SF = 0 or SF = 1 with a ≥ 1-point decrease in MMS SF from baseline; RB = 0), endoscopic remission (ES = 0 or ES = 1, excluding friability), and histologic remission (Geboes scoring of ≤2B.0 [Geboes subscores of 0 for grades 2 b (lamina propria neutrophils), 3 (neutrophils in epithelium), 4 (crypt destruction), 5 (erosion or ulceration)]). The impact of mirikizumab on DC remained consistent despite using the alternative DC which is stringently defined by including biomarker fCal normalization and DC.

Our results suggest that DC was successfully achieved with mirikizumab treatment in up to 16% of patients at W12 and 36% of patients at W52. The consistent achievement of DC across various study stages—induction, maintenance, and open-label extension—indicates the drug’s sustained efficacy. Additionally, achievement of this outcome irrespective of prior biologic treatment implies the potential effectiveness of mirikizumab in patients regardless of treatment experience as both bio-failed and bio-naïve treated patients showed a significant response compared to those with placebo at W52. Patients who achieved DC at W12 showed a higher likelihood of achieving other clinical outcomes at W52. Likewise, achievement of DC at W52 demonstrated an association with achievement of other clinical outcomes at W104.

In this analysis of LUCENT-1 data, the univariable analysis identified left-sided colitis/proctitis, lower fCal level, lower CRP level, baseline MMS, lower ES, lower SF subscore, and no prior biologic or tofacitinib failure as candidate prognostic factors for patients achieving DC at W12. Interestingly, in the LUCENT-2 study only baseline disease location was significantly associated with DC status at W52. This may be attributed to the randomized withdrawal design, in which only mirikizumab responders entered the LUCENT-2 trial. Additionally, while this study, and others, demonstrated that histologic remission was associated with positive clinical outcomes, other studies have not found the same relationship.^13^ The reason for this discrepancy remains unclear, however, it may result from factors such as the retrospective nature of the study design, variability in histologic scoring reproducibility, differences in patient population or disease states, and the timing of treatment and/or disease longevity. Future work will be needed to clearly dissect the role that endoscopic and histologic remission plays in the resolution of UC.

From multivariable analysis, lower CRP level, baseline MMS, lower ES, lower SF subscore, and no prior biologic or tofacitinib failure were significant prognostic factors for achieving DC at W12 among patients receiving mirikizumab. Although this study did not identify factors that were predictive of mirikizumab efficacy over placebo, the multivariable analyses suggest that less severe disease inflammatory activity at baseline is associated with mirikizumab patients achieving DC at W12.

To date, clinical remission and endoscopic mucosal healing are the main treatment targets in patients with UC, while DC (symptomatic, endoscopic, and histologic remission) has more recently been proposed as the ultimate goal in the treatment of UC.^14^ The formal definition of DC, however, is still variable and not established by the medical community. Different definitions of symptomatic and endoscopic-histologic mucosal healing for DC have been suggested based on variable degrees of endoscopic healing (ES = 0 or ES = 1) and with/without inclusion of fCal. Other criteria have been consistent across several definitions of DC, including the requirement for reduced SF and no RB, as well as histologic remission with a Geboes score ≤2B.0, which is aligned with the European Crohn’s and Colitis Organization’s criteria of absence of neutrophils in the mucosa and absence of ulcers or erosions.^15^^,^^16^

In one multicenter, retrospective, cohort study, DC was defined as concurrent clinical (partial-Mayo score ≤2), endoscopic (endoscopic-Mayo score = 0), and histological (Nancy index = 0) remission. By this definition, patients with UC who had DC had a significantly lower risk for future hospitalization and surgery.^17^ In the VARSITY trial, however, DC was defined as a composite outcome with the following criteria, which are less stringent than the definition used in this present analysis: (1) clinical remission, partial Mayo score ≤2 and no individual subscore >1 (excluding sigmoidoscopy subscore); (2) endoscopic improvement, endoscopic subscore ≤1, and (3) absence of active histologic disease (minimum histological disease activity: Robarts Histology Index <5). At W52, 29.2% and 16.3% of patients in the 2 treatment groups had achieved DC.^18^ Meanwhile, in the ongoing VERDICT trial, DC was defined as achievement of a corticosteroid-free Mayo rectal bleeding subscore of 0 plus endoscopic improvement (Mayo Endoscopic Score ≤1) and histologic remission (Geboes score <2B.0). At W16, 34% of patients enrolled in the target group (corticosteroid-free symptomatic remission with endoscopic improvement and histologic remission) had achieved this target.^19^ Although a concept for “comprehensive disease control” has been proposed by Schreiber et al., including rectal bleeding, stool frequency, bowel urgency, abdominal pain, extraintestinal manifestations, disease-related quality of life (QoL), endoscopy, histological inflammatory activity, inflammatory biomarkers, use of corticosteroids, fatigue, and sleep disturbance, this disclosure focuses on the definitions of DC described above.^20^ Future disclosures may address “comprehensive DC” if appropriate data is available. Together, these trials show that DC is a very high bar to achieve, which is reflected by the overall low percentage of patients able to reach it. In our analysis, an alternative definition of DC included the concurrent achievement of symptomatic remission, HEMR, and fCal <250 μg/g, which is a more difficult endpoint to achieve than DC alone. As expected, DC and aDC results were aligned, as fCal is a surrogate for endoscopic and histological disease activity in UC even though thresholds may vary.

This analysis reveals that a significant number of patients achieved DC after 1 year of treatment with mirikizumab, and approximately 1 out of 2 patients attained this outcome after 2 years. This gradual increase in DC rates over time indicates a potential cumulative benefit of continued mirikizumab treatment. Additionally, the association between DC and the improvement of PROs is particularly noteworthy, suggesting that the clinical benefits of mirikizumab lead to tangible enhancements in patients’ QoL, a critical aspect of long-term treatment success. Finally, early achievement of DC by W12, which is associated with better long-term clinical outcomes, is encouraging; this implies that early responders to mirikizumab could anticipate more favorable long-term results, which healthcare providers should consider when making treatment decisions. Achievement of DC was not significantly associated with BU remission, indicating that BU may involve additional unique underlying mechanisms or correlates.

This analysis reveals evidence with DC that surpasses the current standard, supporting a more comprehensive approach to treating UC. The study demonstrates the effectiveness of mirikizumab in achieving DC, promoting a treatment goal. Furthermore, it establishes a clear connection between DC and improved clinical outcomes and PROs. This highlights the potential of mirikizumab to alleviate symptoms and alter the disease course, providing hope for patients seeking an enhanced QoL. Overall, the data support that mirikizumab is effective in achieving and sustaining DC in patients and has the potential for long-term benefits. The study promotes a more complete remission goal, demonstrating the need for a formal definition of DC to ensure consistency in clinical trials, improve comparability of outcomes, and support clinical decision-making.^1^

It is important to consider the limitations of the study. First, to date, there is no formal definition of DC. Within this disclosure 2 definitions are assessed and as discussed other definitions have been proposed by others. As all current definitions for DC use various combinations of clinical outcomes, studies specifically designed to formally define DC would greatly support both clinical trial design and, ultimately, clinical practice. Additionally, the association of early DC and later achievement of endoscopic remission, histologic remission, HEMR, and other clinical outcomes may be inherently correlated because those endpoints are DC components. Ideally, a prospective study assessing DC would be useful in providing a formal definition of DC. Interestingly, at W52 in the placebo group, 19.6% of patients achieved DC (Figure 1B), while 16.8% achieved aDC (Figure S4B), This relatively high PBO response rate was also observed in other primary and secondary outcome measures during LUCENT-1 and LUCENT-2 trials. A possible explanation for this is prior exposure to mirikizumab in the induction phase (Weeks 0-12).^7^^,^^21–23^ NRI is a conservative method used to handle missing data in clinical trials by classifying all patients with missing outcomes as nonresponders. While this approach can prevent overestimation of treatment effects, it may also underestimate efficacy, particularly when missing data are unrelated to treatment failure, potentially introducing bias and reducing statistical power. Last, as the study lacks multiplicity as a clinical trial it may not represent the broader real-world patient population.

## 5. Conclusion

Mirikizumab-treated patients are able to achieve and sustain disease clearance, improving patient outcomes over both short- and long-term treatment periods.

## Supplementary Material

jjaf124_Supplementary_Data

## Data Availability

Eli Lilly and Company provides access to all individual participant data collected during the trial, after anonymization, with the exception of pharmacokinetic or genetic data. Data are available to request 6 months after the indication studied has been approved in the US and EU and after primary publication acceptance, whichever is later. No expiration date of data requests is currently set once data are made available. Access is provided after a proposal has been approved by an independent review committee identified for this purpose and after receipt of a signed data sharing agreement. Data and documents, including the study protocol, statistical analysis plan, clinical study report, blank or annotated case report forms, will be provided in a secure data sharing environment. For details on submitting a request, see the instructions provided at www.vivli.org.

## References

[1] D’Amico F , Magro F, Siegmund B, et al Disease clearance as a new outcome in ulcerative colitis: a systematic review and expert consensus. Inflamm Bowel Dis. 2024;30:1009-1017. 10.1093/ibd/izad159 37549104

[2] Ramos L , Teo-Loy J, Barreiro-de Acosta M. Disease clearance in ulcerative colitis: setting the therapeutic goals for future in the treatment of ulcerative colitis. Front Med (Lausanne). 2022;9:1102420. 10.3389/fmed.2022.1102420 36698823 PMC9868775

[3] Hassan SA , Kapur N, Sheikh F, et al Disease clearance in ulcerative colitis: a new therapeutic target for the future. World J Gastroenterol. 2024;30:1801-1809. 10.3748/wjg.v30.i13.1801 38659483 PMC11036494

[4] Andronic AM , Toader E. P398 Disease clearance: a potential target for management of patients with ulcerative colitis. J Crohn’s Colitis. 2023;17:i529-i529. 10.1093/ecco-jcc/jjac190.0528

[5] Nascimento C , Réves J, Ramos LR, et al P406 Disease clearance in patients with ulcerative colitis treated with aminosalicylates. J Crohn’s Colitis. 2022;16:i398-i398. 10.1093/ecco-jcc/jjab232.533

[6] Bryant RV , Costello SP, Schoeman S, et al Limited uptake of ulcerative colitis “treat-to-target” recommendations in real-world practice. J Gastroenterol Hepatol. 2018;33:599-607. 10.1111/jgh.13923 28806471

[7] D’Haens G , Dubinsky M, Kobayashi T, et al; LUCENT Study Group. Mirikizumab as induction and maintenance therapy for ulcerative colitis. N Engl J Med. 2023;388:2444-2455. https://www.nejm.org/doi/full/10.1056/NEJMoa2207940 37379135 10.1056/NEJMoa2207940

[8] Magro F , Pai RK, Kobayashi T, et al Resolving histological inflammation in ulcerative colitis with mirikizumab in the LUCENT induction and maintenance trial programmes. J Crohns Colitis. 2023;17:1457-1470. 10.1093/ecco-jcc/jjad050 37057827 PMC10588772

[9] Sands BE , D’Haens G, Clemow DB, et al Two-year efficacy and safety of mirikizumab following 104 weeks of continuous treatment for ulcerative colitis: results from the LUCENT-3 open-label extension study. Inflamm Bowel Dis. 2024;30:2245-2258. 10.1093/ibd/izae024 38459910 PMC11630283

[10] D’Haens G , Higgins PDR, Peyrin-Biroulet L, et al Extended induction and prognostic indicators of response in patients treated with mirikizumab with moderately to severely active ulcerative colitis in the LUCENT trials. Inflamm Bowel Dis. 2024;30:2335-2346. 10.1093/ibd/izae004 38271613 PMC11630349

[11] Kamper SJ , Maher CG, Mackay G. Global rating of change scales: a review of strengths and weaknesses and considerations for design. J Man Manip Ther. 2009;17:163-170. 10.1179/jmt.2009.17.3.163 20046623 PMC2762832

[12] Abraham B , Wu J, Vermeire S, et al Efficacy and safety of mirikizumab in the treatment of moderately to severely active ulcerative colitis regardless of baseline modified Mayo score: results from the phase 3 LUCENT trials. Crohns Colitis 360. 2025;7:otaf002. 10.1093/crocol/otaf002 40260308 PMC12010086

[13] Narula N , Aruljothy A, Alshahrani A-A, et al Histologic remission does not offer additional benefit for ulcerative colitis patients in endoscopic remission. Aliment Pharmacol Ther. 2020;52:1676-1682. 10.1111/apt.16147 33131108

[14] Danese S , Roda G, Peyrin-Biroulet L. Evolving therapeutic goals in ulcerative colitis: towards disease clearance. Nat Rev Gastroenterol Hepatol. 2020;17:1-2. 10.1038/s41575-019-0211-1 31520081

[15] Raine T , Bonovas S, Burisch J, et al ECCO guidelines on therapeutics in ulcerative colitis: medical treatment. J Crohns Colitis. 2022;16:2-17. 10.1093/ecco-jcc/jjab178 34635919

[16] Magro F , Doherty G, Peyrin-Birulet LP, et al ECCO position paper: harmonization of the approach to ulcerative colitis histopathology. J Crohns Colitis. 2020;14:1503-1511. 10.1093/ecco-jcc/jjaa110 32504534

[17] D’Amico F , Fiorino G, Solitano V, et al Ulcerative colitis: impact of early disease clearance on long-term outcomes—a multicenter cohort study. United European Gastroenterol J. 2022;10:775-782. 10.1002/ueg2.12288 PMC9486490 36107109

[18] Danese S , Schreiber S, Loftus Jr. Jr et al P271 evolving targets in ulcerative colitis: defining disease clearance in the VARSITY study. J Crohn’s Colitis. 2021;15:S305-S305. 10.1093/ecco-jcc/jjab076.396

[19] Jairath V , Zou G, Adsul S, et al DOP11 Disease clearance after 16 weeks of treatment with vedolizumab in patients with moderate to severe ulcerative colitis: an interim analysis from the VERDICT trial. J Crohn’s Colitis. 2024;18:i92-i93. 10.1093/ecco-jcc/jjad212.0051

[20] Schreiber S , Danese S, Dignass A, et al Defining comprehensive disease control for use as a treatment target for ulcerative colitis in clinical practice: international Delphi consensus recommendations. J Crohns Colitis. 2024;18:91-105. 10.1093/ecco-jcc/jjad130 37586038 PMC10821705

[21] Risankizumab in patients with moderately to severely active ulcerative colitis in the phase 3 INSPIRE and COMMAND studies. Gastroenterol Hepatol (N Y). 2024;20:11-13. https://pubmed.ncbi.nlm.nih.gov/39193121/PMC11345978 39193121

[22] Risankizumab induction therapy in patients with moderately to severely active ulcerative colitis: efficacy and safety in the randomized phase 3 INSPIRE study. Gastroenterol Hepatol (N Y). 2023;19:9-10. https://pubmed.ncbi.nlm.nih.gov/38445187/PMC10910380 38445187

[23] Sands BE , Sands MD, Sandborn WJ, et al; UNIFI Study Group. Ustekinumab as induction and maintenance therapy for ulcerative colitis. N Engl J Med. 2019;381:1201-1214. https://www.nejm.org/doi/full/10.1056/NEJMoa1900750 31553833 10.1056/NEJMoa1900750

[24] Irvine EJ. Development and subsequent refinement of the inflammatory bowel disease questionnaire: a quality-of-life instrument for adult patients with inflammatory bowel disease. J Pediatr Gastroenterol Nutr. 1999;28:S23-S27. https://pubmed.ncbi.nlm.nih.gov/10204520/10204520 10.1097/00005176-199904001-00003

