# Supplementary material for: Mirikizumab impact on disease clearance in patients with moderately to severely active ulcerative colitis: analysis of a pre-specified LUCENT trial endpoint
Source: J Crohns Colitis. 2025 Jul 13;19(9):jjaf124. doi: 10.1093/ecco-jcc/jjaf124 (PMC12561001; doi:10.1093/ecco-jcc/jjaf124)
Supplement: jjaf124_Supplementary_Data [file jjaf124_supplementary_data.zip › Supplementary Material.pdf]

Supplementary Table 1. Baseline characteristics of patients achieving or not achieving DC at Week 12.

	Achieved DC **(N=160)**	Did not achieve DC **(N=1002)**
Age (years), mean (SD)	41.1 (13.2)	42.8 (14.0)
Female, n (%)	75 (46.9)	392 (39.1)
BMI (kg/m^2), mean (SD)	24.7 (5.6)	24.9 (5.3)
Underweight (<18.5 kg/m^2)	11 (6.9)	72 (7.2)
Normal (≥18.5 and <25 kg/m^2)	93 (58.1)	507 (50.6)
Overweight (≥25 and <30 kg/m^2)	33 (20.6)	279 (27.8)
Obese and extremely obese (≥30 kg/m^2)	23 (14.4)	144 (14.4)
Disease duration (years), mean (SD)	7.0 (7.2)	7.1 (6.7)
Disease location		
Proctitis	2 (1.3)	6 (0.6)
Left-sided colitis	114 (71.3)	618 (61.7)
Pancolitis	44 (27.5)	377 (37.7)
Baseline fecal calprotectin (µg/g), median (Q1, Q3)	1336.0 (509.0, 2755.0)	1553.0 (638.0, 3236.0)
Baseline C-reactive protein (mg/L), median (Q1, Q3)	2.4 (0.9, 6.2)	4.5 (1.6, 10.3)
Baseline modified Mayo score, mean (SD)	6.3 (1.3)	6.5 (1.3)
Endoscopic Mayo subscore, n (%)		
Moderate disease (2)	81 (50.6)	306 (30.6)
Severe disease (3)	79 (49.4)	695 (69.4)
Baseline stool frequency, n (%)	5 (3.1)	30 (3.0)
Normal number of stools for subject (0)		
1 to 2 stools more than normal (1)	23 (14.4)	118 (11.8)
3 to 4 stools more than normal (2)	72 (45.0)	281 (28.0)
5 or more stools than normal (4)	60 (37.5)	573 (57.2)
Rectal bleeding subscore, n (%)		
No blood seen (0)	14 (8.8)	135 (13.5)
Streaks of blood with stool less than half the time (1)	48 (30.0)	335 (33.4)
Obvious blood (more than just streaks) or streaks of blood with stool most of the time (2)	86 (53.8)	476 (47.5)
Blood alone passed (3)	12 (7.5)	56 (5.6)
Prior UC therapy, n (%)		
Biologic or tofacitinib exposure	44 (27.5)	455 (45.4)
Biologic or tofacitinib failure	40 (25.0)	439 (43.8)
Anti-TNF failure	38 (23.8)	384 (38.3)
Vedolizumab failure	12 (7.5)	206 (20.6)
Tofacitinib failure	4 (2.5)	36 (3.6)

	Achieved DC **(N=160)**	Did not achieve DC **(N=1002)**
Baseline UC therapy, n (%)		
Corticosteroids	58 (36.3)	406 (40.5)
Immunomodulators	33 (20.6)	247 (24.7)
Aminosalicylates	124 (77.5)	739 (73.8)

BMI, body mass index; DC, disease clearance; SD, standard deviation; TNF, tumor necrosis factor; UC, ulcerative colitis.

Supplementary Table 2. Univariable logistic regression analysis of association between patient demographics and baseline characteristics and DC at Week 12 of LUCENT-1 (NRI) in patients randomized to mrikizumab 300 mg IV in LUCENT-1.

Baseline parameter	Category	N	Univariable analysis	
			OR (95% CI)[a]	p-value[b]
Age	≥40 years vs <40 years	868	0.76 (0.53, 1.09)	0.1346
Sex	Female vs male	868	1.37 (0.95, 1.97)	0.0917
Geographic region	Europe vs North America<br>Other vs North America	868	1.14[c] (0.65, 2.02)<br>1.27[c] (0.74, 2.18)	0.6682
Duration of UC	<1 year vs ≥7 years<br>≥1 to <3 years vs ≥7 years<br>≥3 to <7 years vs ≥7 years	868	1.35 (0.73, 2.51)<br>1.22 (0.77, 1.94)<br>1.04 (0.66, 1.66)	0.7088
Fecal calprotectin	>250 µg/g vs ≤250 µg/g	739	0.51 (0.29, 0.91)	0.0218*
C-reactive protein	>6 mg/L vs ≤6 mg/L	857	0.43 (0.28, 0.66)	<0.0001*
Prior biologic or tofacitinib failure	Not failed vs failed	868	2.60 (1.72, 3.94)	<0.0001*
Corticosteroid use	No vs yes	868	1.34 (0.92, 1.96)	0.1272
Immunomodulator use	No vs yes	868	1.38 (0.88, 2.17)	0.1574
Baseline modified Mayo score	N/A	868	0.83 (0.72, 0.95)	0.0075*
Disease location	Pancolitis vs left-sided colitis[c]	868	0.63 (0.43, 0.94)	0.0245*
Endoscopic Mayo subscore	Moderate disease (2) vs severe disease (3)	868	2.37 (1.64, 3.42)	<0.0001*
Stool frequency Mayo subscore	<3 vs 3	868	2.51 (1.72, 3.66)	<0.0001*
Rectal bleeding Mayo subscore	<2 vs ≥2	868	0.72 (0.50, 1.05)	0.0871

* p value ≤ 0.05

[a] OR indicates quantum of change in response variable due to change of level (categorical) or one unit (continuous) in predictor variable.

[b] p-values were determined from the Wald test for overall predictor effect.

[c] Odds Ratios calculated for each level of response compared to a reference category

Patients with proctitis were combined with those with left-sided colitis when examining disease location.

CI, confidence interval; DC, disease clearance; IV, intravenous; N/A, not applicable, NRI, nonresponder imputation; OR, odds ratio; UC, ulcerative colitis.

Supplementary Table 3. Multivariable logistic regression analysis of association between patient demographics and baseline characteristics and DC at Week 12 of LUCENT-1 (NRI) in patients randomized to mrikizumab 300 mg IV in LUCENT-1.

Baseline parameter	Category	Multivariable analysis	
		OR (95% CI)[a]	p-value[b]
Fecal calprotectin	>250 µg/g vs ≤250 µg/g	0.83 (0.44, 1.54)	0.5458
C-reactive protein	>6 mg/L vs ≤6 mg/L	0.56 (0.34, 0.90)	0.0180*
Prior biologic or tofacitinib failure	Not failed vs failed	2.03 (1.27, 3.25)	0.0030*
Baseline modified Mayo score		1.31 (1.02, 1.68)	0.0320*
Disease location	Pancolitis vs left-sided colitis[c]	0.78 (0.50, 1.24)	0.2963
Endoscopic Mayo subscore	Moderate disease (2) vs severe disease (3)	2.14 (1.30, 3.52)	0.0028*
Stool frequency Mayo subscore	<3 vs 3	3.69 (2.05, 6.63)	<.0001*

* p value ≤ 0.05
[a] OR indicates quantum of change in response variable due to change of level (categorical) or one unit (continuous) in predictor variable.
[b] p-values were determined from the Wald test for overall predictor effect.
[c] Patients with proctitis were combined with those with left-sided colitis when examining disease location.
CI, confidence interval; DC, disease clearance; IV, intravenous; NRI, nonresponder imputation; OR, odds ratio.

Supplementary Figure 1. Study design of LUCENT-1, LUCENT-2, and LUCENT-3.

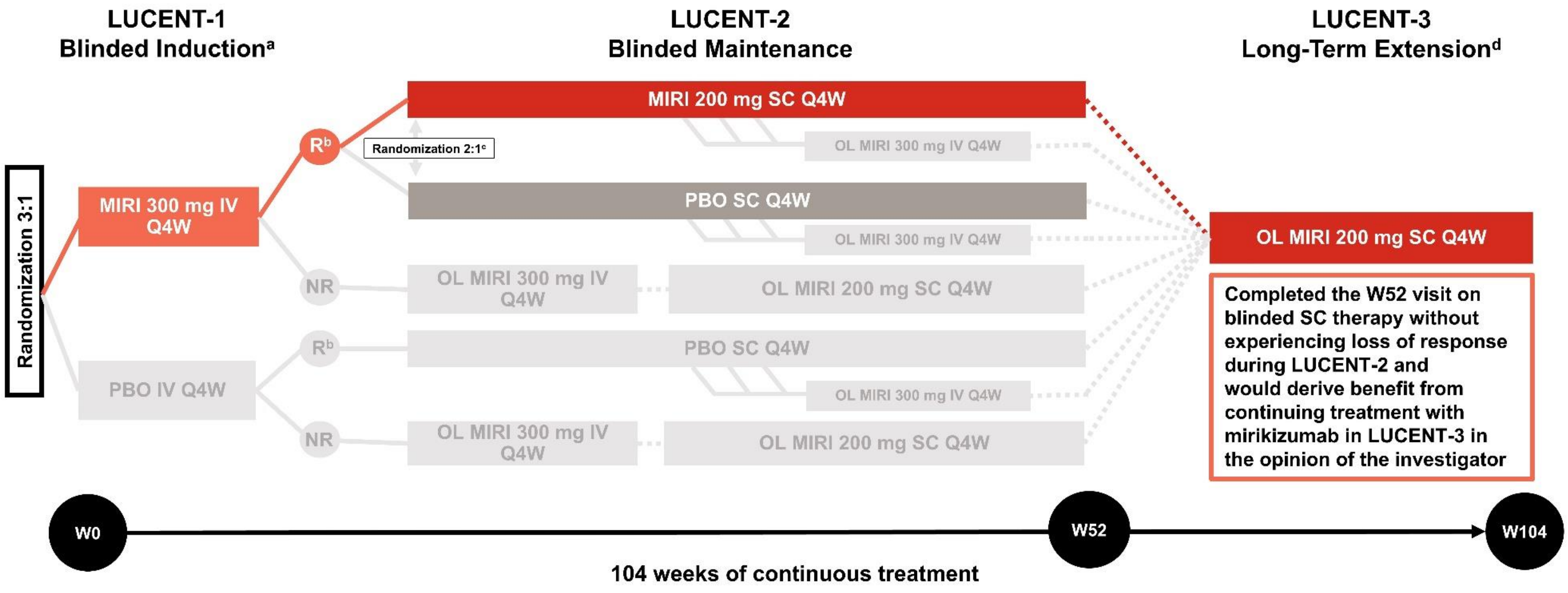

[a] Double-blind, randomized, withdrawal maintenance study in patients who responded to MIRI induction therapy in LUCENT-1.

[b] Clinical responders at W12 were defined as those with a ≥2-point and ≥30% decrease in MMS from baseline with RB of 0 or 1 or a ≥1-point decrease from baseline.

[c] Randomization in LUCENT-2 was stratified by induction remission status, biologic failure status, baseline corticosteroid use, and region.

[d] Figure does not show the full LUCENT-3 program; only the first 52 weeks are presented here. MIRI responders at the end of LUCENT-2 who were eligible for LUCENT-3 were defined as those who completed the W52 visit on blinded MIRI or PBO without loss of response, those who completed the W52 visit on OL MIRI, or those who received IV rescue doses of MIRI after experiencing loss of response and were, in the opinion

of the investigator, considered to be receiving clinical benefit from MIRI therapy. Patients who completed participation in the phase 2 I6T-MC-AMAC study were also eligible for enrollment, and patients from future MIRI UC studies may also be eligible for LUCENT-3.

IV, intravenous; MIRI, mirikizumab; MMS, modified Mayo Score; NR, non-responders; OL, open-label; PBO, placebo; Q4W, every 4 weeks; R, responder; RB, rectal bleeding; SC, subcutaneous; UC, ulcerative colitis; W, Week.

Supplementary Figure 2. For the trial population with baseline modified Mayo Score of 5-9 (excluding 4), (A) Proportion of patients achieving DC at W12 in LUCENT-1. (B) Proportion of patients achieving DC at W40 in LUCENT-2 (52-week treatment in total). (C) Proportion of patients achieving DC at W104 among patients who achieved clinical remission at W52 and subsequently entered the LUCENT-3.

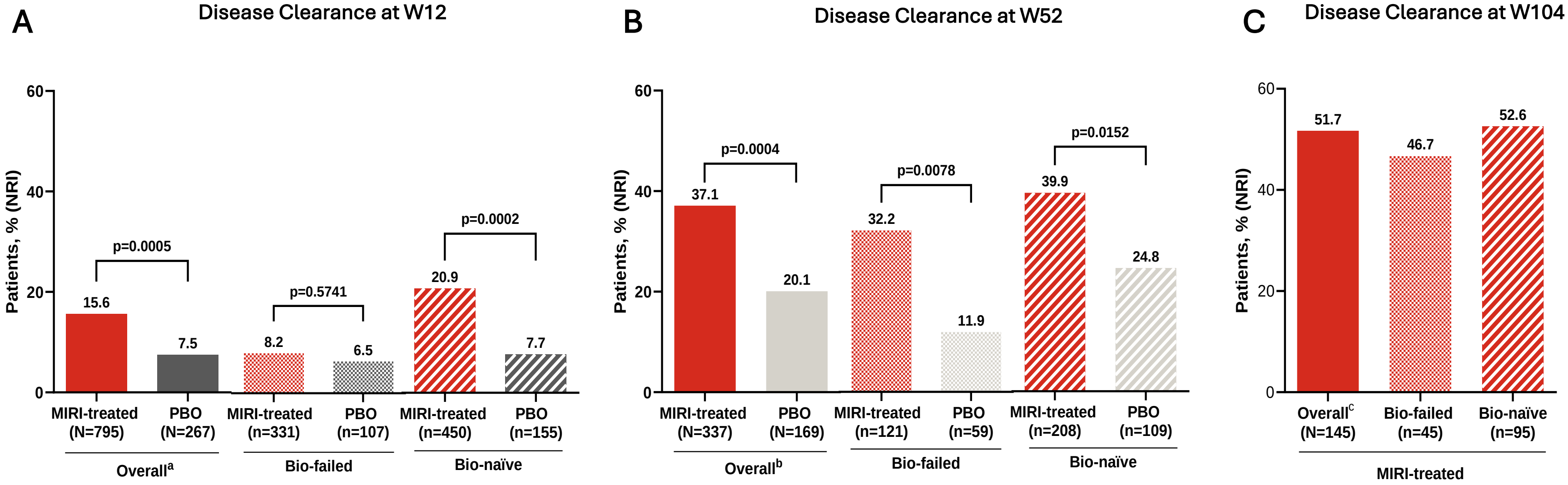

[a] Five patients receiving PBO and 14 patients receiving MIRI were previously exposed to but had no biologic or JAK inhibitor failure.

[b] One patient receiving PBO and 8 patients receiving MIRI were previously exposed to but had no biologic or JAK inhibitor failure.

[c] Five patients were previously exposed to but had no biologic or JAK inhibitor failure.

DC = symptomatic remission + histologic-endoscopic mucosal remission. DC, disease clearance; JAK, Janus kinase; NRI, nonresponder imputation; MIRI, mirikizumab; PBO, placebo; W, Week.

Supplementary Figure 3. Proportion of mirikizumab treated patients achieving DC at Week 104 in Week 52 clinical responders.

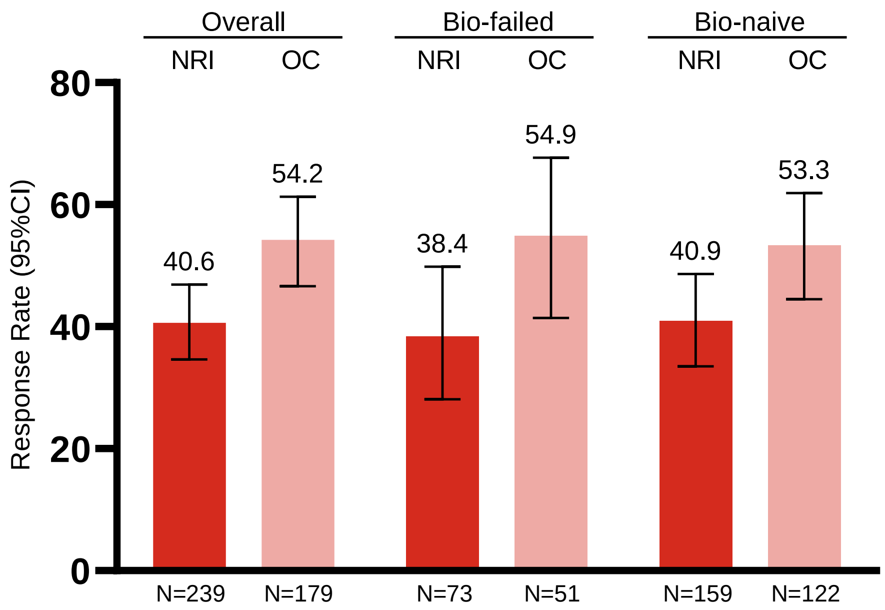

CI, confidence interval; DC, disease clearance; NRI, nonresponder imputation; OC, observed case.

Supplementary Figure 4. The proportion of patients achieving alternative DC at W12 (A) and W52 (B) in the LUCENT trials.

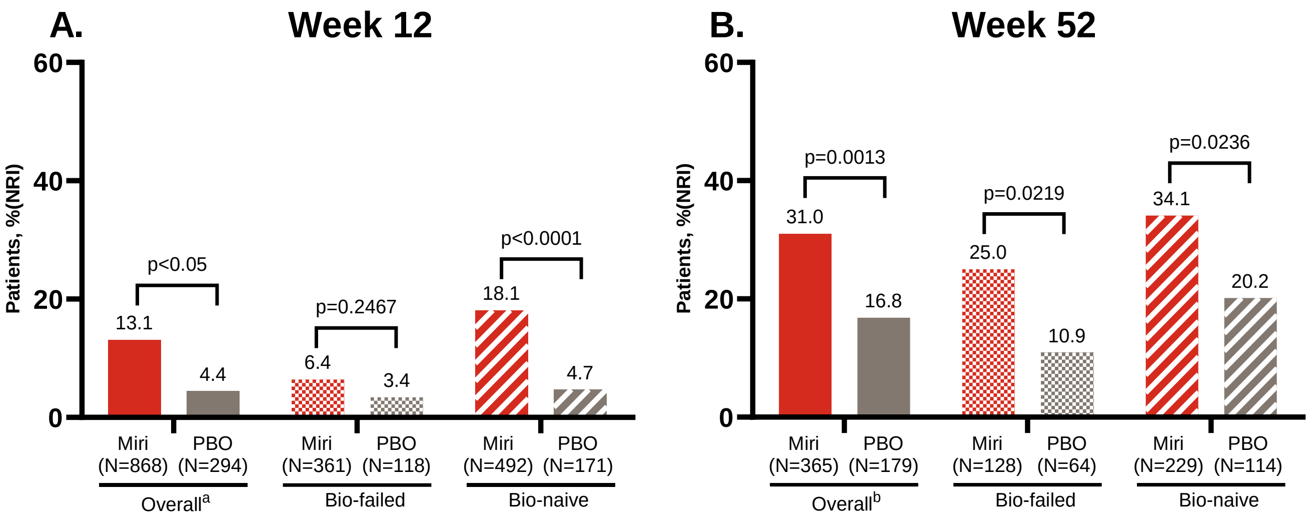

[a] 5 patients receiving PBO and 15 patients receiving Miri were previously exposed to but had no biologic or JAK inhibitor failure.

[b] 1 patient receiving PBO and 8 patients receiving Miri were previously exposed to but had no biologic or JAK inhibitor failure.

DC, disease clearance; JAK, Janus kinase; NRI, nonresponder imputation; MIRI, mirikizumab; PBO, placebo.
